# Inclusion of hazardous drinking does not improve the SCORE performance in men from Central and Eastern Europe: the findings from the HAPIEE cohorts

**DOI:** 10.1186/1471-2458-14-1187

**Published:** 2014-11-20

**Authors:** Olga Vikhireva, Ruzena Kubinova, Sofia Malyutina, Andrzej Pająk, Galina Simonova, Martin Bobak, Hynek Pikhart

**Affiliations:** Epidemiology and Public Health Department, University College London, London, UK; National Institute of Public Health, Prague, Czech Republic; Institute of Internal and Preventive Medicine, Siberian Branch of the Russian Academy of Medical Sciences, Novosibirsk, Russia; Novosibirsk State Medical University, Novosibirsk, Russia; Department of Epidemiology and Population Sciences, Institute of Public Health, Faculty of Health Sciences, Jagiellonian University Medical College, Krakow, Poland

**Keywords:** Cardiovascular mortality and risk factors, SCORE scale, Hazardous drinking, Central and Eastern Europe, Former Soviet Union

## Abstract

**Background:**

The SCORE (Systematic COronary Risk Evaluation) scale uses conventional risk factors for the prediction of the 10-year risk of fatal atherosclerotic cardiovascular disease (CVD). The high-risk version of SCORE is recommended by the European Society of Cardiology for use in the populations of Central and Eastern Europe and former Soviet Union (CEE/FSU). Given the role of hazardous alcohol consumption as an important determinant of CVD mortality in CEE/FSU men, this study investigated whether adding hazardous drinking characteristics to the high-risk SCORE improves its prognostic performance in contemporary population-based male CEE/FSU cohorts.

**Methods:**

The HAPIEE (Health, Alcohol, and Psychosocial factors In Eastern Europe) study follows Czech (seven towns), Polish (Krakow), and Russian (Novosibirsk) cohorts from 2002–2005. In HAPIEE men (n = 8,927), 264 atherosclerotic cardiovascular deaths were registered over the median follow-up time of 6.2-8.1 years.

**Results:**

In HAPIEE men, the baseline levels of the high-risk SCORE ≥5% significantly predicted fatal CVD. After controlling for the high-risk SCORE, binge drinking (drinking ≥100 g of ethanol at least once a month) and problem drinking (≥2 positive answers to CAGE questionnaire) were inconsistently associated with fatal CVD. No marked improvement in calibration and discrimination was observed for the high-risk SCORE extended by these hazardous drinking indicators, and all values of integrated discrimination improvement were <0.5%.

**Conclusions:**

Extending the high-risk SCORE by hazardous drinking parameters failed to improve its prognostic performance across male CEE/FSU population samples. Our findings tentatively support the use of the original high-risk SCORE in male CEE/FSU populations. More research is needed on the potential use of hazardous drinking in cardiovascular risk prediction.

## Background

The SCORE (Systematic COronary Risk Evaluation) scale is a widely used instrument for assessing the risk of future cardiovascular disease (CVD) across European populations [1]. SCORE uses the information on age, gender, blood lipids, blood pressure (BP), and smoking in order to estimate the 10-year risk of cardiovascular mortality in 40-65-year-olds without manifested CVD. Two versions of SCORE were created for European countries with high and low background risk of atherosclerotic CVD mortality – the high-risk one and the low-risk one, respectively. The European Society of Cardiology (ESC) recommends applying the high-risk SCORE to the populations of Central and Eastern Europe (CEE) and former Soviet Union (FSU) [2], even though this version was developed without reference to local data.

Recently, there have been ongoing attempts to improve the SCORE prognostic performance by adding resting heart rate [3], high-density lipoprotein cholesterol [4], and other factors [5, 6] to the model based on conventional risk determinants. Across CEE/FSU populations, which still face high levels of CVD mortality [7], male hazardous alcohol consumption is one of the potential candidates for inclusion in cardiovascular risk models, together with conventional risk factors [7–10]. Although several standardised instruments for the hazardous drinking assessment exist, the prognostic performance of alcohol-extended CVD risk scales has never been examined in CEE/FSU.

Our study aimed to investigate whether the high-risk SCORE calibration and discrimination in men improve after extension by hazardous drinking characteristics, using the individual-level data from three contemporary population-based CEE/FSU cohorts.

## Methods

### Study population and samples

The HAPIEE (Health, Alcohol, and Psychosocial factors In Eastern Europe) study is an ongoing multi-centre study of CVD and other chronic conditions in CEE/FSU [11]. It follows random population samples from the Czech Republic (Havířov/Karviná, Hradec Králové, Jihlava, Kroměříž, Liberec, and Ústí nad Labem), Poland (Krakow), Russia (Novosibirsk), and Lithuania (Kaunas) since the baseline assessment (2002–2005; 2006–2008 in Kaunas) for cause-specific mortality and non-fatal CVD. As the Lithuanian cohort entered the study later and had fewer CVD deaths, it was excluded from the present analyses. The numbers of sampled and examined male subjects, baseline examination years, and response rates are presented in Table 1. At baseline, a questionnaire survey and a physical examination, with a fasting venous blood sample collection, were performed. Mortality data were obtained from national (the Czech Republic) and local (Poland and Russia) registers [11]. The adjudication process for CVD deaths in HAPIEE was extensive, combining data from death certificates with information from CVD registers, autopsy records, and other sources [11]. In the present analyses, the follow-up time was until the end of 2011 for the Czech Republic and the end of 2010 for Poland and Russia, or the date of death, or the date of last contact for those lost to follow-up. The percentage of participants lost to follow-up was lowest in Russia (1%) and highest in Poland (5%), mainly due to withdrawal from the study.

**Table 1:** **Sample selection**

	***Czech Republic***	***Poland***	***Russia***
Baseline, year (response rate, %)	2002-2005 (55)	2002-2005 (61)	2002-2005 (61)
Recruited, N	4,124	5,230	4,269
Within the study age range, N	4,077	5,230	4,264
No pre-existing CVD, N	3,405	3,986	3,254
No missing SCORE values, N	2,659	3,456	3,246
No missing values of SCORE and binge and problem drinking, N	2,517	3,196	3,214

As SCORE predicts cardiovascular risk in individuals over 40 and without pre-existing atherosclerotic CVD [1], we excluded subjects aged <40 at baseline and those with medical evidence or a self-reported history of doctor-diagnosed myocardial infarction, angina, or stroke. In Czech and Polish samples, the study questionnaire was completed at home, prior to medical examination in a clinic. This explains the smaller proportion of Czech and Polish participants with non-missing data. Relatively few women reported hazardous drinking, therefore, only men were included in these analyses. Overall, 8,927 men had available data on baseline SCORE levels and hazardous drinking characteristics (Table 1).

The HAPIEE protocol was approved by the University College London/University College London Hospital Ethics Committee (London, UK) and by local ethics committees at each study centre: the National Institute of Public Health Ethics Committee (Prague, the Czech Republic), the Jagellonian University Ethics Committee (Krakow, Poland), and the Institute of Internal and Preventive Medicine Ethics Committee (Novosibirsk, Russia) [11]. All participants provided written informed consent.

### Measurements

The SCORE risk predictors include age, sex, smoking status, total cholesterol (TC), and systolic BP (SBP). The measurement of these parameters in HAPIEE participants is described in detail elsewhere [11, 12]. Individuals currently and regularly smoking at least one cigarette per day were classified as current smokers; never and ex-smokers were considered non-smokers, according to the SCORE criteria [1]. SBP and TCH measurement was subjected to extensive quality control.

Our additional risk factors of interest were binge drinking and problem drinking (positive CAGE score) [11]. Annual alcohol consumption and drinking patterns in HAPIEE were estimated by the graduated frequency (GF) method [13, 14], which assesses how often during the past 12 months more than a specific amount of alcohol (approximately 0.5 drinks, 1–2, 3–4, 5–6, 7–9, or ≥10 drinks) was consumed; the frequency is measured on a nine-point scale, ranging from “never” to “every day”. Based on the GF data, we defined male binge drinking as consumption of ≥100 g of ethanol per drinking session at least once a month. The presence of alcohol-related problems in the last 12 months was assessed by the CAGE questionnaire [15], which asked whether the person had felt he/she should cut down on drinking; whether people had annoyed the respondent by criticising his/her drinking; whether the person had felt bad or guilty about his/her drinking; and whether he/she had a drink first thing in the morning. Participants with ≥2 positive answers were considered problem drinkers.

In line with SCORE end-points [1], the study outcome was atherosclerotic cardiovascular death (International Classification of Diseases 10 codes: I10-I15, I20-I25, I44-I73 (except I45.6, I51.4, I52, I60, I62, I67.1, I67.5 and I67.7), R96.0, and R96.1).

### Statistical analyses

We used the high-risk SCORE version, which is recommended by the ESC for CEE/FSU populations [2], to predict the risk of fatal atherosclerotic CVD in all male HAPIEE samples. The recently introduced Czech and Polish SCORE versions were not used, as they lack a detailed description of their development and recalibration and are very similar to the original high-risk SCORE scale [16, 17].

The prognostic performance of risk prediction scales, such as SCORE, is assessed via calibration and discrimination [18, 19]. Calibration shows how close the predicted and observed risks are. Discrimination demonstrates how accurately the participants who will experience events (such as fatal CVD) during the follow-up are separated from those who will remain event-free. Better calibration and discrimination are denoted, respectively, by lower χ^2^ values and higher *p*-values in the Hosmer-Lemeshow test [20] and higher values of the Harrell’s C-statistic [21]. The additional prognostic value of extra risk predictors could be assessed in likelihood ratio tests (LRT). Lower LRT *p*-values signify more pronounced differences between the nested baseline and extended models and, therefore, better predictive performance of the latter. Recently introduced and more clinically relevant parameters of risk reclassification are net reclassification index and integrated discrimination improvement (IDI). As our additional risk factors were dichotomized and, hence, specific to HAPIEE samples, we used IDI, which is relatively independent of risk cut-offs and categories and reflects the ability of the extended model to improve average sensitivity without compromising average specificity [22, 23].

We performed our analyses of the prognostic ability of the extended high-risk SCORE separately in each sample. Our extended models included hazardous drinking parameters and SCORE values calculated with the high-risk SCORE chart (an equivalent of assessing drinking patterns of an individual after calculating their SCORE risk levels). A similar approach was used in some other studies of the extended SCORE [5, 6], and it was demonstrated that the models with calculated SCORE values and those with fitted SCORE variables perform similarly [5]. In our analyses, we first explored the role of high-risk SCORE, binge drinking, and problem drinking as cardiovascular mortality predictors, using Cox regression models. Then we estimated calibration and discrimination of the high-risk SCORE extended by hazardous drinking parameters, calculating Hosmer-Lemeshow χ^2^, Harrell’s C, LRT *p*, and IDI. The baseline Model 1 (high-risk SCORE only) was compared to Model 2 (high-risk SCORE and binge drinking), Model 3 (high-risk SCORE and problem drinking), and Model 4 (high-risk SCORE, binge drinking, and problem drinking). We were aware that the relatively small number of atherosclerotic CVD deaths in Czech and Polish samples could affect the prognostic performance of the extended high-risk SCORE. Therefore, the results for these samples should be interpreted with caution.

The use of Cox proportional hazards regression models was justified by the high *p*-values in Schoenfeld’s test. The competing-risk regression analyses [24], which take into account the risk of death from causes other than atherosclerotic CVD, produced very similar results (not presented) to those of standard Cox analyses. No significant interactions between the high-risk SCORE and hazardous drinking indicators were detected. In HAPIEE samples, alcohol consumption frequency demonstrated a J- or U-shaped association with fatal CVD (not presented). The available HAPIEE data did not permit the differentiation between never-drinkers and ex-drinkers, and excluding all self-reported never-drinkers would substantially reduce the sample size. Therefore, our analyses focused on binge and problem drinking. Simultaneously extending the high-risk SCORE by these two variables was possible, due to low values of phi correlation coefficient (not presented). To enable comparisons between non-extended and extended models, the analyses included only subjects with known values of SCORE and hazardous drinking characteristics. The high-risk SCORE calibration was affected by the fact that the current follow-up of HAPIEE samples is less than 10 years (Table 2). However, our focus was on the calibration changes after the high-risk SCORE extension by binge and problem drinking, rather than on SCORE calibration *per se*. As the Hosmer-Lemeshow test quantifies the agreement between predicted and observed events across risk deciles, it was applied to the non-dichotomised high-risk SCORE, which treats individual levels of absolute risk (percentages in the respective SCORE chart cells) as a continuous variable. All statistical analyses were performed using Stata/IC 12.0 (StataCorp LP, Texas, USA).

**Table 2:** **Sample characteristics**

	***Czech Republic***	***Poland***	***Russia***
N	2,517	3,196	3,214
Mean age (SD), years	57.9 (7.1)	57.1 (6.9)	57.5 (7.0)
Age groups, %			
45-49	17.4	20.6	18.9
50-54	20.0	21.4	21.3
55-59	20.8	21.8	21.6
60-64	21.3	18.5	18.2
65+	20.6	17.8	20.0
Current smoking, %	26.6	33.1	51.2
Mean TC (SD), mmol/l	5.7 (1.0)	5.8 (1.1)	6.0 (1.2)
Mean SBP (SD), mm Hg	143.7 (18.5)	141.6 (20.2)	141.6 (22.6)
Binge drinking, %	18.1	9.7	33.0
Problem drinking, %	10.0	9.2	20.8
Median follow-up (IQR), years	8.1 (7.7-8.9)	7.1 (6.9-7.7)	6.2 (5.7-7.0)
Atherosclerotic cardiovascular deaths, N (%)	60 (2.4)	55 (1.7)	159 (5.0)

IQR, interquartile range; SBP, systolic blood pressure; SD, standard deviation; TC, total cholesterol.

## Results

### Description of the study samples

The characteristics of male HAPIEE samples are presented in Table 2. At baseline, the mean age of participants was close to 57 years, with relatively similar sizes of the five-year age groups. Smoking prevalence was higher in Russian men (>50%), compared to Czech and Polish men (<35%). Mean levels of TC were close to 6 mmol/l in all samples. The highest mean SBP levels (144 mm Hg) were observed for Czech men. The hazardous drinking prevalence in Russian men was twice as high as in their Czech and Polish counterparts. Despite the shortest follow-up period, the percentage of atherosclerotic CVD deaths in Russian men (5%) was higher than that in Czech and Polish men (2%).

### SCORE performance and hazardous drinking

In all HAPIEE samples, the baseline high-risk SCORE ≥5% significantly predicted the risk of CVD mortality over the subsequent 6.2-8.1 years, both before and after adjustment for hazardous drinking (adjusted HR 3.6-8.6; Table 3). Due to the current follow-up being under 10 years, the sample-specific numbers of CVD deaths were relatively low, and 95% confidence intervals were wide. There was little evidence that either parameter of hazardous drinking is a significant predictor of fatal CVD after adjustment for high-risk SCORE. In Polish men, problem drinking appeared a significant predictor of fatal CVD even after adjustment for high-risk SCORE and binge drinking, but this sample had the lowest number of atherosclerotic cardiovascular deaths (n = 55).

**Table 3:** **Prognostic performance of the high-risk SCORE before and after inclusion of binge and problem drinking**

	***Czech Republic***	***Poland***	***Russia***
**Model 1** (SCORE only)			
SCORE HR (95% CI)	8.64 (3.13-23.82)	3.50 (1.71-7.14)	7.00 (3.69-13.28)
Hosmer-Lemeshow χ^2^ (*p*)^a^	9.21 (0.33)	5.98 (0.54)	46.71 (<0.01)
Harrell’s C	0.6586	0.6262	0.6276
**Model 2** (SCORE and binge drinking)			
SCORE HR (95% CI)	8.56 (3.11-23.62)	3.50 (1.71-7.15)	6.99 (3.69-13.27)
Binge drinking HR (95% CI)	0.74 (0.35-1.55)	1.36 (0.62-3.01)	1.15 (0.83-1.59)
Hosmer-Lemeshow χ^2^ (*p*)^a^	11.07 (0.20)	7.57 (0.48)	38.61 (<0.01)
Harrell’s C	0.6694	0.6321	0.6404
LRT *p*	0.40	0.46	0.40
IDI, % *(p)*	0.039 (0.32)	0.013 (0.68)	0.043 (0.27)
**Model 3** (SCORE and problem drinking)			
SCORE HR (95% CI)	8.62 (3.12-23.76)	3.57 (1.75-7.30)	7.00 (3.69-13.28)
Problem drinking HR (95% CI)	0.64 (0.23-1.77)	2.34 (1.18-4.65)	1.21 (0.84-1.74)
Hosmer-Lemeshow χ^2^ (*p*)^a^	8.82 (0.36)	14.85 (0.06)	38.27 (<0.01)
Harrell’s C	0.6674	0.6525	0.6364
LRT *p*	0.36	0.03	0.32
IDI, % *(p)*	0.053 (0.15)	0.220 (0.09)	0.041 (0.33)
**Model 4** (SCORE, binge drinking, and problem drinking)			
SCORE HR (95% CI)	8.56 (3.10-23.61)	3.60 (1.75-7.30)	7.00 (3.69-13.27)
Binge drinking HR (95% CI)	0.79 (0.37-1.71)	0.98 (0.41-2.32)	1.10 (0.77-1.56)
Problem drinking (95% CI)	0.69 (0.24-1.97)	2.36 (1.12-4.97)	1.16 (0.79-1.72)
Hosmer-Lemeshow χ^2^ (*p*)^a^	11.68 (0.17)	15.20 (0.06)	38.86 (<0.01)
Harrell’s C	0.6705	0.6611	0.6443
LRT *p*	0.54	0.09	0.53
IDI, % *(p)*	0.071 (0.13)	0.219 (0.09)	0.060 (0.21)

CI, confidence interval; IDI, integrated discrimination improvement; HR, hazard ratio; LRT, likelihood ratio test. ^a^Calculated for continuous high-risk SCORE.

Hosmer-Lemeshow χ^2^ values were relatively low for the original high-risk SCORE, with Russian men as the only exception. The high-risk SCORE extension by binge or problem drinking only slightly reduced, or even increased, χ^2^ values and, therefore, demonstrated either minimal or no calibration improvement.

For all samples, the inclusion of binge and problem drinking in the high-risk SCORE resulted in a modest increase in Harrell’s C-statistics. This increase was the most pronounced in Polish men (from 0.63 for Model 1 to 0.66 for Model 4). All LRT *p*-values were relatively high: the lowest ones were observed in Polish men (0.03; Model 3). The degree of discrimination improvement, quantified by IDI, was below 0.5% in all samples. Overall, calibration and discrimination of the original, non-extended high-risk SCORE appeared to be similar, or only marginally worse, compared to the performance of the high-risk SCORE extended by hazardous drinking indicators.

## Discussion

To the best of our knowledge, this is the first attempt to investigate the prognostic performance of the high-risk SCORE extended by hazardous drinking in CEE/FSU male population samples. The high-risk SCORE significantly predicted atherosclerotic cardiovascular mortality in HAPIEE men, both before and after adjustment for hazardous alcohol consumption. On the other hand, adding binge and problem drinking to the high-risk SCORE did not substantially improve its calibration and discrimination, which supports the use of the original, non-extended instrument.

### Strengths and limitations

Several methodological issues should be considered when interpreting our results. First, although HAPIEE samples are not representative of the whole countries, they are the best available CEE/FSU sources of individual-level cohort data on the levels of cardiovascular risk factors and fatal CVD in the 2000s.

Second, as in most studies, HAPIEE participants were likely to be healthier than non-responders to the baseline survey and those lost to follow-up. This possible discrepancy could be enhanced by the complete case analyses and dilute the association of interest. The available multiple imputation methods employ the assumption of data missing (completely) at random [25, 26], which was unlikely to be met in our samples. In addition, the Cox regression results across the samples with the highest SCORE missingness were similar for complete and multiply imputed data (not presented). Therefore, the possible selection bias due to non-response and SCORE missingness was unlikely to substantially affect the strength of the association between cardiovascular risk factors and mortality.

Third, binge drinking and problem drinking are only two parameters out of the wide range of alcohol consumption characteristics which potentially influence cardiovascular risk. On the other hand, the comparability, validity, and reliability of binge and problem drinking as hazardous alcohol consumption parameters have been previously demonstrated for male HAPIEE populations [27]. The self-reported measures of hazardous drinking, despite underestimating the actual consumption, satisfactorily reflect the ranking of participants in terms of their drinking behaviour. Their inclusion in the high-risk SCORE was justified by the findings of several previous studies on heavy or hazardous drinking as an important cause of cardiovascular mortality in CEE/FSU [8–10].

Fourth, across HAPIEE samples, it was not feasible to differentiate between never- and ex-drinkers, as well as between participants who could never be considered as hazardous drinkers and those with previous history of hazardous drinking. This might have reduced the strength of the association between current hazardous alcohol consumption and fatal CVD in HAPIEE men. At the same time, the evidence from the subsample of Novosibirsk men suggested that their drinking patterns were sufficiently stable over a six-year period [8].

Finally, the baseline levels of conventional and additional risk factors could have changed during the follow-up period and, therefore, result in potential regression dilution bias, or underestimation of the association of interest [28]. However, the general concept of risk prediction implies focusing on the current exposure levels, in order to estimate the future risk of the outcome.

### Consistency with other studies

The validity of our findings, despite the potential limitations mentioned above, is supported by the general agreement between the levels of major risk factors and CVD mortality in HAPIEE samples and the respective national cross-sectional estimates and trends presented in the WHO Global InfoBase [29] and WHO systematic reviews [30–32].

Across all HAPIEE samples, the high-risk SCORE remained a significant predictor of CVD death after accounting for binge and problem drinking. We are unaware of any published CEE/FSU or Western studies adjusting the effects of SCORE by alcohol consumption parameters. The absence of consistent, significant associations between hazardous drinking and fatal CVD (negative and mixed results for binge and problem drinking, respectively) in HAPIEE men disagrees with the findings of several previous studies in Russian men [8–10]. However, alcohol was not associated with increased CVD risk among participants of the Russian Lipid Research Clinics Study [33] and men from St. Petersburg [34] and Arkhangelsk [35].

The potential explanations of the absence of consistent alcohol effects in our study include the relatively short follow-up time, low numbers of atherosclerotic CVD deaths in the currently available data (particularly in the Czech and Polish samples), and the potential changes in drinking behavior over time. Multiple mechanisms of adverse cardiovascular effects of hazardous alcohol consumption involve conventional risk factors, such as blood pressure and cholesterol [36]. Controlling for these factors, which are included in SCORE, might over-adjust the association between hazardous drinking and fatal CVD. In addition, the magnitude of the alcohol effects on cardiovascular risk could be markedly influenced by recent episodes of hazardous drinking, and, hence, be larger in case–control vs. cohort studies [9, 10]. Moreover, the exact mechanisms underlying the link between hazardous drinking and atherosclerotic vs. non-atherosclerotic fatal CVD remain unclear. It has been suggested that in Russia, alcohol-related non-atherosclerotic CVD (such as alcoholic cardiomyopathy leading to fatal arrhythmias and heart failure) and acute alcohol poisoning could potentially be misclassified as acute ischemic heart disease, and this may drive the association between alcohol and cardiovascular mortality [10, 37–39]. The regional and temporal variability in the relative impact of such misclassification on fatal CVD rates could explain, at least partly, the mixed findings on the role of alcohol as the determinant of CVD in CEE/FSU. In this context, the present results should be regarded as lacking clear evidence of an association between hazardous drinking and cardiovascular mortality, rather than proving the absence of an association.

In our study, adding hazardous drinking parameters did not substantially improve calibration or discrimination of the high-risk SCORE. We were unable to identify any CEE/FSU or Western studies which have investigated the prognostic performance of the alcohol-extended SCORE or another cardiovascular risk scale. Moreover, no existing cardiovascular risk scale includes any drinking parameters.

## Conclusion

To summarise, the prospective individual-level Czech, Polish, and Russian data from three large population samples have shown that the high-risk SCORE significantly predicts CVD mortality in men both before and after adjustment for hazardous drinking characteristics. At the same time, the improvement in calibration and discrimination of the extended high-risk SCORE has been minimal. The implications of these findings can be summarised as follows. First, our results tentatively support the use of the original high-risk SCORE in the male CEE/FSU populations. However, due to the mixed evidence on the association between hazardous alcohol consumption and fatal CVD, further research is needed on hazardous drinking as a CVD risk factor, particularly in CEE/FSU. Future studies may show that other measures (such as surrogate/non-beverage drinking), when added to SCORE separately or in combination, could improve the SCORE prognostic performance in specific populations. Second, there is a growing interest in the use of extended risk models among individuals at intermediate risk [16, 40, 41]. The latest ESC guidelines [2], published after our findings were obtained, recommend the novel biomarker measurement and cardiovascular imaging among asymptomatic adults at “moderate risk” (SCORE ≥1% and <5%). Therefore, it is important to investigate whether additional risk factors provide clinically and statistically significant improvement in the SCORE performance among people with intermediate risk levels. Finally, our findings have confirmed the key role of conventional risk factors. Their control at both the population and individual levels should reduce the CVD burden in CEE/FSU and prevent the reversal of declining CVD rates in other populations [2, 42, 43].
